# Retrospective Cohort Analysis of Class II Human Leukocyte Antigen (HLA) Alleles in Children With Steroid-Dependent Nephrotic Syndrome Treated With Cyclophosphamide

**DOI:** 10.7759/cureus.76245

**Published:** 2024-12-23

**Authors:** Sehrish Javed, Aasia Zubair, Habib Qaiser, Ali Asghar A Lanewala, Khawar Abbas, Wajiha Musharraf

**Affiliations:** 1 Pediatric Nephrology, Sindh Institute of Urology and Transplantation, Karachi, PAK; 2 Immunology, Sindh Institute of Urology and Transplantation, Karachi, PAK

**Keywords:** association, cyclophosphamide, hla class ii antigens, sdns, treatment outcomes

## Abstract

Background

The role of specific human leukocyte antigen (HLA) alleles as a risk factor for susceptibility, protection, and response to cyclophosphamide (CYC) treatment has been studied in patients with idiopathic nephrotic syndrome (INS). This study investigates the association of class II HLA alleles and the treatment outcome in children with steroid-dependent nephrotic syndrome (SDNS) who were treated with CYC.

Methods

A total of 77 children who were diagnosed with SDNS and had received CYC at least a year before were enrolled. After obtaining informed consent from the parents, blood samples were collected to type the HLA class II locus (DR and DQ) using sequence-specific primers (SSP). An equal number of adult healthy controls (AHC) were included.

Results

The median age of the study participants at disease onset was five (IQ: 3-7) years with a male-to-female ratio of 1.48:1. The common HLA alleles found in the study cohort and controls were DRB1*7 45 (29%), DRB1*15 30 (19.5%), and DQB1*2 57 (37%), and DRB1*15 31(20%), DRB1*17 36 (23%), DQB1*6 42 (27%), and DQB1*2 38 (25%), respectively. Identification of a particular allele to predict a good or poor response was not statistically significant (p-value >0.05).

Conclusion

The study demonstrates the common HLA alleles in the cohort. However, a specific allele that can predict a good and poor response to CYP was not identified. Further large-scale, prospective multicenter studies are needed to identify such alleles to decide the use of CYC in the SDNS population judiciously.

## Introduction

The incidence of childhood idiopathic nephrotic syndrome (INS) varies from 1.15 to 16.9 per 100,000 children in different geographical regions and ethnic backgrounds [1]. Most children respond to steroids and are classified as steroid-sensitive nephrotic syndrome (SSNS). Approximately half of these children experience frequent relapses and are further classified as frequently relapsing nephrotic syndrome (FRNS) or SDNS [2]. Cyclophosphamide (CYC), an alkylating agent, is preferred for the treatment of SDNS due to its proven efficacy in maintaining prolonged remission [1-4].

Although the pathophysiology of INS is still unclear, genetic, immunological, and environmental factors are implicated, resulting in podocyte dysfunction and proteinuria [5]. The human leukocyte antigen (HLA) is implicated in increasing the susceptibility to both the onset and relapse of INS [6]. The polymorphism in the HLA loci varies across the population, resulting in differences in genetic and susceptibility patterns [7].

The candidate gene approach was utilized initially to identify HLA alleles. Recently, genome-wide association studies (GWAS) have provided better insights into HLA loci identification using single nucleotide polymorphisms (SNP). Several studies have identified the role of HLA in both susceptibility and protection to INS. Specifically, HLA B12 and HLA DR7 are reported to be associated with poor responses to CYC [8].

Limited studies exist on the association of HLA phenotypes with treatment response to CYC in children with SDNS. The side effects of CYC are feared and include bone marrow suppression, malignancy, and the likelihood of infertility. Therefore, it would be essential to identify which children can benefit from CYC treatment. We aim to investigate if HLA alleles are linked with the treatment response to the alkylating agent and if specific HLA alleles are associated with post-CYC outcomes. CYC can then be used judiciously if a better response to CYC can be anticipated in most of the SDNS children.

## Materials and methods

The study was approved by the Institutional Ethical Review Committee (SIUT-ERC-2023/A-463). After obtaining informed consent from the parents and assent from adolescent children, they were enrolled in the study over a period of six months, from 1st December 2023 to 31st May 2024. Blood samples were collected for HLA typing by a trained phlebotomist using a standard sampling technique. A standard proforma was used to collect demographic and clinical details of the enrolled participants, especially the clinical parameters like serum albumin, blood pressure (BP), and the time to respond to steroids at first presentation were also recorded.

All the children who were under regular follow-up and had received CYC in standard dosing for SDNS before December 2022 were identified from the medical records and included in the study. Patients with infantile or adolescent nephrotic syndrome, primary or secondary steroid-resistant nephrotic syndrome (SRNS) before receiving CYC, and patients who did not receive a cumulative dose of 168 mg/kg were excluded from the study. The adult healthy controls (AHC), who were living kidney donors for end-stage kidney disease (ESKD) patients and whose HLA was routinely tested as part of the transplant workup, were randomly included in the study from the records of the Department of Immunology for comparison with the patient population.

Standard definitions for SDNS, FRNS, infrequently relapsing nephrotic syndrome (iFRNS), and secondary SRNS were used [3]. A good response to CYP was defined as sustained remission maintained for more than one year after CYP treatment, while a poor response was identified if a child was unable to maintain sustained remission after completing the course [9].

Remission is defined as a negative urine dipstick for protein or a first-morning urinary protein-to-creatinine ratio (UPCR) <0.2 mg/mg. Relapse is defined as +3 proteinuria on the urine dipstick or a first-morning UPCR >2 mg/mg.

HLA typing

HLA class II locus (DR and DQ) were typed using sequence-specific primers (SSP) from the collaborative transplant study (CTS) in Heidelberg, Germany, by polymerase chain reaction (PCR). An equal number of HLA class II (DR and DQ) alleles from AHC were included in the study for comparison. These healthy controls (HC) were the living donors of ESKD patients who received live-related kidney transplants.

Statistical analysis

An open EPI sample size calculator was used for the sample size. Assuming the proportion of patients visiting the OPD in six months is 100 and that the patient proportion will be 50% (50% good and 50% bad response), with a 95% confidence interval and a 5% margin of error, the sample size calculated was 80 using the formula: Sample size, n=(DEFF*Np(1-p))/((d2/Z21-α/2*(N-1)+p*(1-p)). A similar proportion of AHC was taken from the records to compare the HLA of the patient and HC.

All the continuous variables with normal distribution were expressed as mean± standard deviation while the data with skewed distribution was described as median and IQ. The categorical variables were described as percentages. The relationship between continuous, dependent, and independent variables was analyzed using the Chi-square or Fisher exact T-test. To find the association of HLA alleles with response to CYC, OR was calculated using 2x2 contingency tables, and unadjusted ORs were calculated for each allele separately with a confidence interval of 95%. All the statistical analyses were performed using SPSS version 27 (IBM SPSS Statistics, Armonk, NY). The p-value of <0.05 was taken as significant.

## Results

To assess if the response to CYP can be predicted by HLA allele typing, we included 77 SDNS patients in this study. The median age of study participants was five (IQ: 3-7) years, with a slight male predominance (46, 60%). Most of the study population belonged to Urdu 32 (42%), followed by Sindhi 24 (31%) ethnic background. The time to remission of the first episode of INS was two weeks in most of the participants 63 (82%), and normal/elevated BP was seen in 73 (95%). Table 1 demonstrates the demographic and clinical parameters of the study participants.

**Table 1:** Basic demographic and clinical parameters of children and healthy cohort, N=77 CYC, cyclophosphamide; HC, healthy control; BP, blood pressure; CYC, cyclophosphamide; FRNS, frequently relapsing nephrotic syndrome; SRNS, steroid-resistant nephrotic syndrome; MCD, minimal change disease; FSGS, focal segmental glomerulosclerosis

Variable	Value
Median age at disease presentation (years)	5 (IQ: 3-7)
Gender	
Boys	46 (60%)
Girls	31 (40%)
Ethnicity	
Urdu	32 (42%)
Sindhi	24 (31%)
Others	21 (27%)
HC ethnicity	
Urdu	29 (38%)
Sindhi	23 (30%)
Others	25 (32%)
Consanguinity	
Yes	50 (65%)
No	27 (35%)
Time to first remission	
2 weeks	63 (82%)
4 weeks	14 (18%)
BP at presentation	
Normal/elevated BP	73 (95%)
Stage I hypertension	04 (05%)
Albumin at presentation	
1-2 mg/dL	51 (66%)
>2 mg/dL	26 (34%)
Cholesterol at presentation	
180-360 mg/dL	50 (65%)
361-520 mg/dL	27 (35%)
Response to CYC	
Good	30 (39%)
Poor	47 (61%)
Histopathology	
MCD	24 (31%)
FSGS	11 (14%)
IgMN	13 (17%)
Biopsy not required	29 (38%)
Post-CYC outcomes	
Sustained remission	12 (15.5%)
iFRNS	19 (25%)
FRNS	05 (6.5%)
Secondary SRNS	41 (53)

We divided our study cohort into good and bad responders according to the response to CYC. A total of 30 (39%) of our study participants demonstrated a good response to CYC, maintaining sustained remission post-CYC. However, 47 (61%) did not respond well and became FRNS (5, 6.5%) and secondary SRNS (41, 53%) after CYC therapy.

We compared CYC response to clinical parameters like age at presentation, gender, serum albumin, cholesterol, BP at presentation, and remission of the first episode. We found a statistically significant association between a good response to CYC and the time to first remission, as well as normal/elevated BP at presentation, using a Pearson Chi-Square test (Χ²: 10.922, p-value: 0.001, and Χ²: 6.448, p-value: 0.04), respectively. However, no statistically significant association was observed between a good response and gender or younger age at presentation (<6 years), with Pearson Chi-Square values of Χ²=0.193 (p=0.660) and Χ²=0.281 (p=0.591), respectively. Table 2 demonstrates factors associated with CYC response.

**Table 2:** Factors associated with CYC response CYC, cyclophosphamide; iFRNS, infrequently relapsing nephrotic syndrome

Variable	CYC responder		P-value
	Good (n=30)	Poor (n=47)	
Gender, boys (n=46)	17 (22%)	29 (38%)	0.66
Age <6 years at presentation	16 (21%)	31 (40%)	0.59
Albumin at presentation	30 (39%)	47 (61%)	0.24
Cholesterol at presentation	30 (39%)	47 (61%)	0.08
Normal/elevated BP at presentation	30 (39%)	43 (56%)	0.04*
Sustained remission + iFRNS	30 (39%)	01 (5.3%)	0.001*

HLA typing and association of HLA in predicting response to CYC

To assess whether a response to CYC can be predicted with the HLA allele, we typed 154 alleles of the study participants and AHC using SSP by PCR. We found DRB1*7 45 (29%), DRB1*15 30 (20%), and DQB1*2 57 (37%) to be the most common alleles among the study population. The common alleles in the AHC were DRB1*15 31(20%), DRB1*17 36 (23%), DQB1*6 42 (27%), and DQB1*2 38 (25%). Table 3 shows HLA alleles and their frequencies.

**Table 3:** HLA DR and DQ alleles of SDNS children and AHC, N=154 Table 3 demonstrates the HLA alleles of the study participants and AHC. The number of alleles typed is 154 for 77 participants. HLA DQB1*4 was present in any of the AHCs. AHC, adult healthy controls; HLA, human leukocyte antigen; SDNS, steroid-dependent nephrotic syndrome

HLA of SDNS children		HLA of HC	
HLA DRB1	Value	HLA DRB1	Value
DRB1*1	05 (03%)	DRB1*1	04 (2.5%)
DRB1*4	15 (10%)	DRB1*4	07 (4.5%)
DRB1*7	45 (29%)	DRB1*7	06 (04%)
DRB1*8	02 (1.5%)	DRB1*9	01 (0.5%)
DRB1*9	02 (1.5%)	DRB1*10	15 (10%)
DRB1*11	25 (16%)	DRB1*11	24 (16%)
DRB1*13	06 (04%)	DRB1*12	02 (01%)
DRB1*14	01(0.5%)	DRB1*13	18 (12%)
DRB1*15	30 (20%)	DRB1*14	10 (6.5%)
DRB1*16	04 (2.5%)	DRB1*15	31 (20%)
DRB1*17	19 (12%)	DRB1*17	36 (23%)
HLA DQB1	Value	HLA DQB1	Value
DQB1*2	57 (37%)	DQB1*2	38 (25%)
DQB1*4	01 (0.5%)	DQB1*5	32 (21%)
DQB1*5	12 (08%)	DQB1*6	42 (27%)
DQB1*6	29 (19%)	DQB1*7	30 (19%)
DQB1*7	29 (19%)	DQB1*8	07(05%)
DQB1*8	16 (10%)	DQB1*9	05 (03%)
DQB1*9	10 (6.5%)	-	-

**Figure 1:**
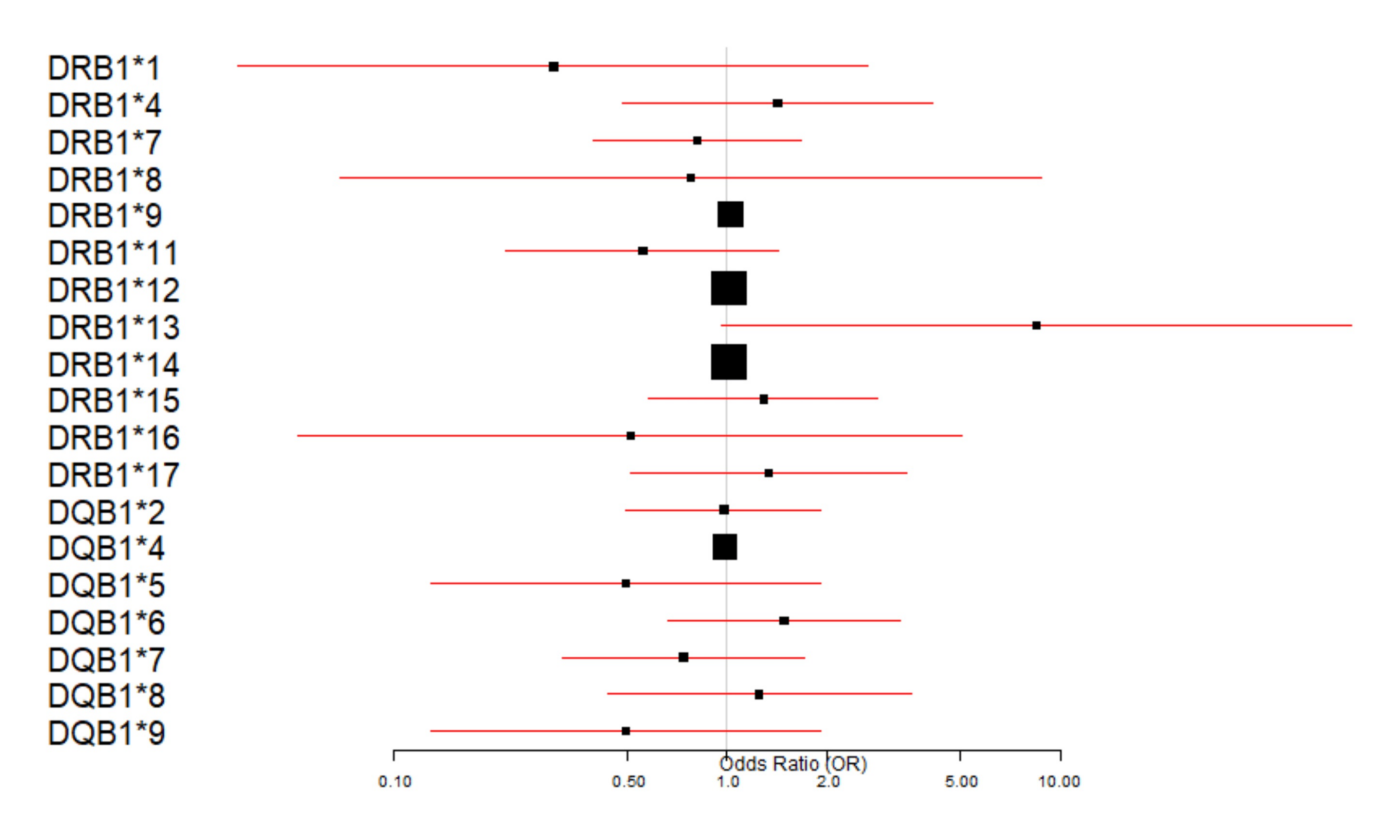
HLA alleles and treatment response The forest plot predicts the association of HLA alleles with the response to CYC. The HLA alleles are on the y-axis, and the OR is on the x-axis. The OR is represented by black squares. It tells the association of alleles with the treatment response (good or bad outcome). OR = 1, there is no association of alleles with the outcome; OR = >1, increased odds of a good outcome; OR = <1, decreased odds of a good outcome. HLA, human leukocyte antigen

We further analyzed whether a risk or protective allele could be identified in the study cohort to guide future CYC treatment in SDNS patients. The analysis revealed no statistically significant results (p-value >0.05) to indicate a protective or risk factor allele. The forest plot in Figure 1 illustrates the association of HLA with the response to CYC treatment.

## Discussion

The exact etiology of childhood INS remains to be elucidated. Advancements in genomics and proteomics have identified multiple phenotypes of this disease. Several clinical parameters, such as age less than 4 years, presence of severe hypoalbuminemia, hematuria, hypertension at initial presentation, and time to respond to initial steroid therapy, have been identified as prognostic factors for SSNS [10]. Similarly, multiple studies have identified specific class II HLA alleles that could predispose children to a variety of phenotypes [11-15]. Our study examined the association of HLA class II alleles (DR and DQ) with SDNS patients treated with CYC.

The median age of the participants at disease onset was five (IQ: 3-7) years. There was a predominance of boys, 46 (60%). Multiple studies corroborate the male predominance found in our study [16-18]. Most of our study participants achieved first remission within two weeks of steroid therapy, 63 (82%). This duration was comparable to the mean time to the first remission in the FR/SDNS group of Lee H et al. [16]. Better responses to CYC were reported by Moorani KN et al. and Dhuria KS et al.; they reported 57.7% and 50% responses to CYP in their cohort, respectively [19,20]. These responses were slightly higher than what we found in our study population, 30 (39%). This difference is likely attributed to the fact that our study cohort was smaller compared to the former studies.

The good responders had earlier times to first remission and normal BP at presentation, which was also found to be statistically significant. However, we could not demonstrate any statistically significant association with clinical and biochemical characteristics like younger age at presentation, gender, hypoalbuminemia, proteinuria, and hypercholesterolemia. This finding is corroborated by Dinçel N et al., who found no prognostic association with treatment response in their cohort [21]. On the contrary, Dhuria KS et al. documented that age less than five years is a good prognostic marker [20].

The exact etiology of SSNs is still not well understood. However, various genes have been identified to be associated with INS [22]. The association of haplotypes DQA1*01:01/DQB1*05:01/DRB1*01:01 with susceptibility of Henoch-Schönlein purpura (HSP) [11], identification of HLA-A30 with primary FSGS by Batal I et al. [14], and demonstration of a strong association of HLA DQA1 with pediatric SSNS [12] are notifiable. However, specific HLA alleles cannot be linked to a certain disease due to polymorphism exhibited by the HLA class II antigens [7].

The HLA DRB1*7 and DQB1*2 were the most common alleles in our study. Jia et al. reported the former allele [17], while the latter allele was reported by Ramanathan et al. [23] in their South Indian cohort. On the contrary, the HLA-A 11:01 allele demonstrated a strong susceptibility to SSNS in the Chinese Han population [16]. These findings can be attributed to the diversity in the genetic makeup of people of different racial backgrounds.

Konrad et al. and Amal et al. have identified that HLA DR7 positivity was associated with a shorter remission period after treatment with alkylating agents [24,25]. Studies from Sri Lanka, South India, and Japan have found different alleles and haplotypes associated with the susceptibility of SSNS or SRNS [17,23,26]. However, none of the latter studies have shown the association of HLA alleles with the treatment response to CYC. Therefore, we analyzed our population to identify a specific allele that can predict a good or bad response to the treatment. We could not identify a particular HLA allele that could have been associated with a good or poor response to CYC in the study cohort. A possible explanation for this could be that these alleles are not associated with cytostatic treatment response and the polymorphic properties of HLA antigens.

An important limitation of the study is the lack of opportunity to include those SDNS patients who maintained complete remission and did not follow-up regularly, in contrast to the poor responders in the study. A retrospective methodology, small sample size, and inability to type other HLA alleles and HLA haplotypes impose certain other limitations to the study.

## Conclusions

To conclude, the study reveals HLA DQB1*2 and DRB1*7 as the most common allele in the study cohort. However, a particular allele that could predict a good response to treatment in these patients was not identified. With the use of advanced HLA genotyping methods, such as GWAS, future large-scale multicenter studies, including SDNS patients and age-matched HC, are needed to determine the association of HLA alleles and haplotypes with the treatment response to steroid-sparing agents.
